# Supplementary material for: ProteinShader: illustrative rendering of macromolecules
Source: BMC Struct Biol. 2009 Mar 30;9:19. doi: 10.1186/1472-6807-9-19 (PMC2672931; doi:10.1186/1472-6807-9-19)
Supplement: Additional file 1 — ProteinShader program without source code. This compressed file contains the complete ProteinShader program including associated libraries, but no source code. A README.txt file gives an overview of the ProteinShader distribution, and the index.html file in the help subdirectory has directions on getting started with the program as well as a set of tutorials. [file 1472-6807-9-19-S1.zip › ProteinShader-beta-0_9_4-binary/help/api/org/proteinshader/gui/listeners/controlpanel/ScalePanelListenerFactory.html]

ScalePanelListenerFactory (ProteinShader API)


|  |  |  |
| --- | --- | --- |
| **Overview** \| **Package** \| **Class** \| **Use** \| **Tree** \| **Deprecated** \| **Index** \| **Help** | |  |
| **PREV CLASS**   **NEXT CLASS** | **FRAMES**    **NO FRAMES**     **All Classes** |  |
| SUMMARY: NESTED \| FIELD \| CONSTR \| METHOD | DETAIL: FIELD \| CONSTR \| METHOD |  |


---


## org.proteinshader.gui.listeners.controlpanel Class ScalePanelListenerFactory

```
java.lang.Object
  org.proteinshader.gui.listeners.controlpanel.ScalePanelListenerFactory
```

---

``` public class ScalePanelListenerFactory extends Object ```

Creates the listeners for the AtomScalePanel.

---

**Constructor Summary**

`ScalePanelListenerFactory(Mediator mediator,
AtomScalePanel scalePanel)`
  
          Constructs a ScalePanelListenerFactory.


| **Method Summary** | |
| --- | --- |
| `ActionListener` | `createApplyButtonActionListener()`             Creates an action listener for the 'Apply' button. |
| `ActionListener` | `createDefaultButtonActionListener()`             Creates an action listener for the 'Default' button. |
| `ChangeListener` | `createSpinnerChangeListener()`             Creates an change listener for a spinner. |

**Methods inherited from class java.lang.Object**

`clone, equals, finalize, getClass, hashCode, notify, notifyAll, toString, wait, wait, wait`

**Constructor Detail**

### ScalePanelListenerFactory

```
public ScalePanelListenerFactory(Mediator mediator,
                                 AtomScalePanel scalePanel)
```

:   Constructs a ScalePanelListenerFactory.

    **Parameters:**: `mediator` - the centralized Mediator object that listeners call on to accomplish their task.: `scalePanel` - the ScalePanel that this class creates listeners for.


**Method Detail**

### createSpinnerChangeListener

```
public ChangeListener createSpinnerChangeListener()
```

:   Creates an change listener for a spinner. This change listener
    has the same effect as the 'Apply' button action listener.

    :   **Returns:**: The action listener.

---


### createApplyButtonActionListener

```
public ActionListener createApplyButtonActionListener()
```

:   Creates an action listener for the 'Apply' button.

    :   **Returns:**: The action listener.

---


### createDefaultButtonActionListener

```
public ActionListener createDefaultButtonActionListener()
```

:   Creates an action listener for the 'Default' button. The listener
    calls on the applyDefaultScale() method of the ScalePanel before
    redrawing the canvas.

    :   **Returns:**: The action listener.


---


|  |  |  |
| --- | --- | --- |
| **Overview** \| **Package** \| **Class** \| **Use** \| **Tree** \| **Deprecated** \| **Index** \| **Help** | |  |
| **PREV CLASS**   **NEXT CLASS** | **FRAMES**    **NO FRAMES**     **All Classes** |  |
| SUMMARY: NESTED \| FIELD \| CONSTR \| METHOD | DETAIL: FIELD \| CONSTR \| METHOD |  |


---

# *Copyright © 2007-2008*
